# 5-Year effectiveness of an oral cholera vaccine in a cholera outbreak in rural Haiti: a case–control study

**DOI:** 10.1016/j.lana.2026.101500

**Published:** 2026-05-30

**Authors:** Louise C. Ivers, Yodeline Guillaume, Allison LaHood, Gertrude C. Augustin, Alain Casseus, Damien M. Slater, Jason B. Harris, Ralph Ternier, Molly F. Franke

**Affiliations:** a Massachusetts General Hospital, Center for Global Health, Boston, MA, USA; b Massachusetts General Hospital for Children, Boston, MA, USA; c Harvard Medical School, Boston, MA, USA; d Harvard Global Health Institute, Cambridge, MA, USA; e Zanmi Lasante, Cange, Haiti; f Harvard T.H. Chan School of Public Health, Boston, MA, USA

**Keywords:** Cholera, Vaccine, Outbreak

## Abstract

**Background:**

The World Health Organization recommends use of killed oral cholera vaccines (kOCV) as part of cholera prevention and control, but data on the duration of effectiveness of the most widely available vaccine, Euvichol® is limited. We evaluated the effectiveness of Euvichol® 5 years after a kOCV campaign conducted in Haiti in 2017.

**Methods:**

We undertook a frequency-matched case–control study in Mirebalais, Haiti between October 2022 and February 2023. Cases were patients with acute watery diarrhea, had a stool sample positive for *V. cholerae* by polymerase chain reaction, and had been eligible for kOCV during the 2017 campaign. Cases were frequency-matched with community controls by age group and neighborhood. Participants reported sociodemographics, risk factors, and vaccination status. We used adjusted logistic regression analyses to estimate vaccine effectiveness (VE) of at least one kOCV dose versus none.

**Findings:**

We analyzed 93 cases and 135 controls. 22% of the cases and 51% of the controls self-reported vaccination. Receiving Euvichol in 2017 was associated with a 5-year adjusted VE of 78% (95% CI: 57%–89%, p < 0.0001).

**Interpretation:**

Receiving any Euvichol® effectively protected against cholera in Haiti, 5 years after a two-dose vaccination campaign. This has important implications for public health planning, especially in considering of the appropriate timing of boosting schedules and comparative effectiveness analyses.

**Funding:**

US National Institute of Allergy and Infectious Diseases of the National Institutes of Health and the Gates Foundation.

## Rezime

**Kontèks:**

Òganizasyon Mondyal Sante (OMS) rekòmande itilizasyon vaksen antikolerik oral inaktive (VCO) nan kad prevansyon ak kontwòl kolera, men done sou dire efikasite vaksen ki pi disponib la, Euvichol®, limite. Nou te evalye efikasite Euvichol® 5 ane apre yon kanpay VCO Ministe Sante Piblik ak Popilasyon te fè an Ayiti an 2017.

**Metòd:**

Nou te fè yon etid ka-temwen aparye an frekans nan Mibalè, Ayiti ant oktòb 2022 ak fevriye 2023. Ka yo te pasyan ki te gen dyare dlo egi, ki te teste pozitif pou V. cholerae pa tès reyaksyon chèn polimeraz, epi ki te elijib pou VCO pandan kanpay 2017 la. Ka yo te aparye ou asosye ak tèmwen kominotè pa tranj laj ak katye. Patisipan yo te rapòte done sosyodemografik, faktè risk, ak estati vaksinal. Nou te itilize analiz regresyon lojistik ajiste pou estime efikasite vaksinal (EV) pou omwen yon dòz VCO kont/pa rapò a okenn dòz.

**Rezilta:**

Nou te analize 93 ka ak 135 temwen. 22% nan ka yo ak 51% nan temwen yo te rapòte yo te vaksine. Resevwa Euvichol® an 2017 te asosye ak yon EV ajiste sou 5 ane ki te egal a 78% (95% IC: 57%-89%, p<0.0001).

**Entèpretasyon:**

Resevwa nenpòt kantite dòz Euvichol® te bay yon proteksyon efikas kont kolera an Ayiti, 5 ane apre yon kanpay vaksinasyon ak de dòz vaksen. Sa gen enplikasyon enpòtan pou planifikasyon sante piblik, espesyalman nan evalyasyon sou kalandriye ki apwopriye pou rapèl vaksinal ak analiz konparatif sou efikasite.

**Finansman:**

Enstiti Nasyonal pou Alèji ak Maladi Enfektyez Etazini nan Enstiti Nasyonal Sante (NIAID, NIH) ak Fondasyon Gates te kontribye nan finansman etid la men yo pa t gen okenn enfliyans sou konsepsyon an, metòd yo, analiz la, oswa atik sa.

**Research in context**

**Evidence before this study**

The Global Taskforce for Cholera Control vaccine working group published a systematic review and meta-analysis of protection from killed whole-cell oral cholera vaccines (kOCVs) in 2025 that included studies published up to March 8, 2024. That review identified five randomized controlled trials (RCTs) and ten observational studies and found that the average two-dose kOCV efficacy from RCTs was 55% at 12 months after vaccination, decreasing to 44% at 48 months. The average two-dose effectiveness from observational studies was 69% after 12 months, waning to 47% after 48 months. Only one study assessed efficacy at five years, with no effectiveness data available beyond four years. In January 2026, we searched PubMed and Web of Science for studies published from March 9, 2024 to January 1, 2026 for any new relevant data on duration of protection of kOCV since the studies included in the meta-analysis. We used the following search terms: cholera∗[Title/Abstract] AND (vaccin∗[Title/Abstract]) AND (effect∗[Title/Abstract] OR efficacy[Title/Abstract] OR protect∗[Title/Abstract]). We screened 74 results and found one additional study, reporting on kOCV effectiveness after two years in Cameroon, that did not add to the evidence on five-year duration of protection.

**Added value of this study**

The last assessment of kOCV protection at five years was conducted over a decade ago with the Shanchol® vaccine in India. Since at least 2024, the Euvichol® vaccine or its derivative formulations have been the main components of the global kOCV stockpile, but data on its long term effectiveness are limited. This vaccine effectiveness study is the first long-term study outside a highly endemic setting for cholera, and the first ‘real world’ evidence showing that any kOCV, specifically Euvichol®, provides protection against medically-attended cholera at five years.

**Implications of all the available evidence**

The available evidence suggests that in an epidemic-prone setting (such as Haiti) as well as in an endemic setting (such as India) two doses of kOCV confers protection against cholera for as long as five years and therefore kOCV can play a role in long-term control of cholera, in addition to other public health measures.

## Introduction

Cholera cases have surged globally since 2021, including in Haiti, where an outbreak began in October 2022 after three years without a confirmed case. The World Health Organization (WHO) recommends the use of killed whole-cell oral cholera vaccines (kOCV) as part of cholera prevention and control, and a stockpile of kOCV is available for countries to use. For some years, the majority of the stockpile has been comprised of Euvichol®, a bivalent kOCV that was pre-qualified by WHO in 2015 after safety and immunogencity studies demonstrated its non-inferiority to Shanchol®, a kOCV with similar components that itself was studied in a double-blind placebo-controlled randomized controlled trial in India, but has recently been out of production.^1^, ^2^, ^3^

Whereas short-term data are available, evidence on the long-term effectiveness of kOCVs in protecting from cholera is limited. One study measured the efficacy of two doses of Shanchol® after 5 years of follow-up and two studies have reported on vaccine effectiveness extending to 48 months.^3^, ^4^, ^5^ Estimates of the duration of protection of vaccines against cholera is important for designing re-dosing (boosting) strategies and for understanding the comparative effectiveness and economic implications of different interventions. Further, because effectiveness of cholera vaccines is likely influenced by incident exposure to *V. cholerae*, the duration of protection of kOCVs across different epidemiologic contexts may vary.^4^^,^^6^ Having previously assessed the two-year vaccine effectiveness of Euvichol® after a mass vaccination campaign in Haiti, we aimed in this study to evaluate its effectiveness at 5 years.^7^

## Methods

### Setting

We conducted this research in Mirebalais, Haiti, beginning in October 2022. We initiated enrollment within weeks of an explosive, large cholera outbreak following a period of almost 3 years without detected cholera cases in the entire country. The study site is a diarrhea treatment unit within the largest hospital in central Haiti, managed by Zanmi Lasante in partnership with the Haitian Ministry of Health. Care is provided free of charge to patients. The hospital is located in the Commune of Mirebalais (population approximately 100,000 people). A cholera epidemic in Haiti first began in 2010 and resurged in 2022 after a period of no reported cases. During the study period (October 19, 2022 to February 10, 2023), 1055 Mirebalais residents attended the treatment unit with suspected cholera.

Five years before this resurgence, a two-dose kOCV campaign using Euvichol® was led by the Haitian Ministry of Health and Population in November and December 2017 in Mirebalais.^6^ According to administrative reporting, 88,377 individuals (90% of a targeted population of 98,563) received a first dose of OCV, and 69,905 individuals (79% of those vaccinated) received a second dose, resulting in an estimated 71% administrative coverage.^8^ In response to the 2022 outbreak, a new single-dose kOCV campaign subsequently took place beginning December 19, 2022.

### Study design

To estimate the effectiveness of vaccination in 2017 against medically-attended cholera in 2022, we conducted a frequency-matched case–control study. Patients presenting with acute watery diarrhea–defined as three or more loose, non-bloody, watery or liquid stools in a 24-h period, with an onset of three days or fewer before presentation–were recruited as potential cases from a diarrhea treatment center in Mirebalais, Haiti, between October 19th and December 19th, 2022.^9^ The last date of enrollment of cases was the launch date of the new single-dose kOCV campaign in the region. Cases were those that tested positive for *V. cholerae* by polymerase chain reaction (PCR) and were eligible for kOCV at the time of the 2017 campaign (i.e., aged ≥12 months and living in the area at the time).

We sought controls from the same neighborhood (called ‘communal section’ in Haiti) as cases. Study workers approached residences close to where a case had occurred, excluding homes within the same compound as a case (a compound known as *lakou* is an enclosed area with a cluster of residential houses for multigenerational families), as we anticipated that uptake of the cholera vaccine was likely to be highly correlated within the *lakou*.^10^ Controls were individuals who reported that they had not experienced acute watery diarrhea during the 2022 outbreak at the time of their interview, but would seek medical treatment if they developed severe illness or diarrhea. Enrollment of controls was intended to happen within two weeks of case enrollment being completed, however due to sociopolitical instability and gang violence that made movement around the countryside risky, we extended enrollment of controls for multiple weeks beyond enrollment of cases. Controls were ultimately enrolled between December 7, 2022 and February 10, 2023. To manage the overlap between the 2022 kOCV campaign timeline and enrollment of controls, including the possibility that controls might receive a vaccine in the 2022 campaign, we undertook a sensitivity analysis restricted to controls enrolled within 10 days of the last enrolled case.

In our analysis, controls were frequency-matched to cases by age group (6–14 years and >15 years) and neighborhood of residence.

We based power calculations on planned enrollment of 70 participants with confirmed cholera and 140 controls. We estimated the frequency of two-dose vaccination among controls to be 50%.^7^ Assuming a Type I error probability of 0.05, we would have 80% power to detect a vaccine effectiveness of 57% or higher (two-sided test). We also conceived of a test-negative design as an alternate methodology, however ultimately an insufficient number of medically-attended diarrhea cases tested negative for cholera, and our frequency matching methodology offered better power.^11^

### Procedures

Trained study workers conducted survey interviews with cases at the diarrhea treatment center and with controls at their homes in their native language of Haitian Creole. All participants reported socio-demographics (e.g., age, sex, household size), risk factors for cholera (e.g., frequency of handwashing), cholera exposure history (e.g., household history of diarrhea and cholera), and cholera vaccination. Oral cholera vaccination status was assessed for both cases and controls by self-report during survey interviews and by study workers’ examination of vaccination cards, when available. To ensure that reported vaccination status corresponded to the 2017 campaign, we conducted further interviews with all subjects in the study to confirm vaccination status using a structured memory aid including questions about personal events that occurred in 2017 to help anchor the year of interest. Participants were also asked to recall certain elements of the vaccination campaign, such as the vaccination post location, activities, and staff. Furthermore, we used visual aids that showed images of the vaccine itself and its oral administration. Adult heads of household provided information, including on vaccination, for minor children.

To confirm incident cholera, we collected fresh stool samples from enrolled cases at the time of presentation for medical evaluation in clean, non-chlorinated disposable containers and transported in a cool box to the medical facility's laboratory for processing and storage. For each case, a portion of the sample was spotted on filter paper (Whatman 903® Protein Saver cards), dried, and stored at ambient temperature in individual bags with desiccant. Samples were transported to our laboratory in Boston, Massachusetts for testing by PCR. We selected the PCR method for diagnosis as it had been shown to be more sensitive than culture in some settings, including ours.^12^^,^^13^ To detect toxigenic *V*. *cholerae*, we predefined a positive PCR test as requiring both ctxA and hlyA at a threshold of 34 cycles or lower to ensure high specificity for clinically meaningful infection. This was based on a prior comparison with data from Liu and colleagues.^14^ Samples with only one target detected or with threshold cycle ≥35 were classified as indeterminate due to low target concentration and reproducibility concerns. A negative call was defined as having no hlyA or ctxA detected.

For PCR testing in the Boston laboratory, fecal sample DNA was prepared from two 6-mm diameter punches of the dried fecal specimens using the ThermoFisher MagMax Microbiome Ultra kit (Waltham, MA, USA) on the KingFisher Flex instrument. Real-time PCR was performed in two independent reactions using sets that target the *V. cholerae* hlyA and ctxA genes using primers and probes obtained from Integrated DNA Technologies (Coralville, IA): hlyA_F, CTGAATGATCAACTCGGTTATCGTC, hlyA_R, TCGATGCGTTAAACACGAAGTG ATAAT, hlyA_P,56-FAM/CGTGAGTGGTCAACCGA TGCGATTGC/3IABkFQ, ctxA_F, CATAGAGCTTGGAGGGAAGAGC, ctxA_R, TCGTCAAGGAAT TTTACACCTAGACT,ctxA_P,56-FAM/AATGCTCCA/ZEN/AGATCATCGAT GAGTAATACTTG//3IABkFQ. Five microliters of template DNA was tested in a 20 μL PCR using 2× iQ Multiplex Powermix (Bio-Rad, Hercules, CA) with primers and probes at a final concentration of 200 nM. PCR runs were performed on an ABI 7500 Fast instrument (Thermo Fisher Scientific, Waltham, MA) with the following conditions: a single 2-min 95 °C hot-start activation step, followed by 40 cycles of 95 °C for 10 s, and 60 °C for 1 min. Positive and negative controls were included with each run. Results were reported as positive, indeterminate, or negative.

### Statistical analysis

Incident medically-attended cholera was the primary outcome, and self-reported receipt of the oral cholera vaccine (at least one dose versus none) in 2017 was the main exposure. We used logistic regression to calculate odds ratios, 95% confidence intervals, and P values. Field effectiveness of the vaccine was calculated using the formula for vaccine efficacy: Vaccine efficacy = (1- relative risk). The relative risk was estimated from multivariable logistic regression analyses adjusted for matching factors and potential confounders identified *a priori* from a list of known cholera risk factors that were differentially distributed across cases and controls (Table 1). These included age (continuous), neighborhood (Grand Boucan; Sarazin; Crête Brûlée or Gascogne), sex (binary), household size (continuous), ever attended school (binary), electricity in the home (binary), history of household diarrhea in the past two weeks (binary), household contact history of spending a night in a cholera treatment unit (binary), frequency of handwashing (categorical), and agriculture as a main income generating activity. We combined Crête Brûlée or Gascogne into a single category due to small numbers in Gascogne. We assessed for nonlinearity of continuous variables on the log odds scale using the Box–Tidwell test. Data were complete for nearly all covariates. A single case was missing data on household history of diarrhea in the two weeks prior to interview; we imputed this value to 0.

**Table 1:** Characteristics of cases and controls in a 5-year study of an oral cholera vaccine effectiveness (Euvichol®) in rural Haiti, Oct–Dec 2022.

Variable	Cases, n (%)	Controls, n (%)
	(N = 93)	(N = 135)
Communal Section		
Grand Boucan	60 (65)	88 (65)
Gascogne	1 (1)	3 (2)
Sarazin	22 (24)	36 (27)
Crête Brûlée	10 (11)	8 (6)
Female	44 (47)	82 (61)
Median age [interquartile range]	23 [9, 38]	27 [12, 43]
Earthen floor in home (versus cement, wood, or other)	32 (34)	51 (38)
Ever attended school	78 (84)	91 (67)
Home has electricity	56 (60)	81 (60)
Median household size [interquartile range]	6 [4, 7]	5 [4, 7]
Agriculture is a main income generating activity	32 (34)	56 (41)
Reported frequency of handwashing (daily)		
None	3 (3)	6 (4)
1	11 (12)	28 (21)
2	32 (34)	32 (24)
3	22 (24)	29 (21)
≥4	25 (27)	40 (30)
Household member with cholera in the last two weeks	6 (6)	4 (3)
Household member ever spent a night in cholera treatment unit	23 (25)	18 (13)
Household member with diarrhea in the last two weeks^a^	17 (18)	10 (7)

a One case had missing data for this variable.

We conducted stratified analyses by age group (aged 1–5 years during the 2017 campaign, versus older than 5 years) adjusting for neighborhood. We conducted a sensitivity analysis restricted to controls enrolled within 10 days of the date of the last enrolled case (in December 19, 2022), thus corresponding to the 10 days following the initiation of the 2022 single-dose kOCV vaccination campaign in the region. This sensitivity analysis was motivated by the fact that most controls were enrolled after the 2022 vaccination campaign began, and 2022 vaccination could increase diarrhea-free person-time and thus the likelihood of being selected as a control. If vaccination in 2022 and 2017 were correlated, 2017 vaccination may be over-represented among controls, potentially leading to an overestimation of 2017 vaccine effectiveness during the 2022 outbreak. We expected this potential bias to be mitigated in an analysis restricted to controls enrolled most closely to the time of cases and prior to the 14-day window typically considered as conferring protection. Data were analyzed using SAS, Version 9.4.

### Ethics approval

We received ethical approval for this study from the Mass General Brigham Institutional Review Board (IRB) in the USA (Protocol # 2018P000350) on Oct, 5, 2022 and the Zanmi Lasante IRB in Haiti (Protocol # ZLIRB13012023). Written informed consent was obtained from all study participants.

### Role of the funding source

The funder of the study had no role in study design, data collection, data analysis, data interpretation, or writing of the report.

## Results

**Fig. 1:**
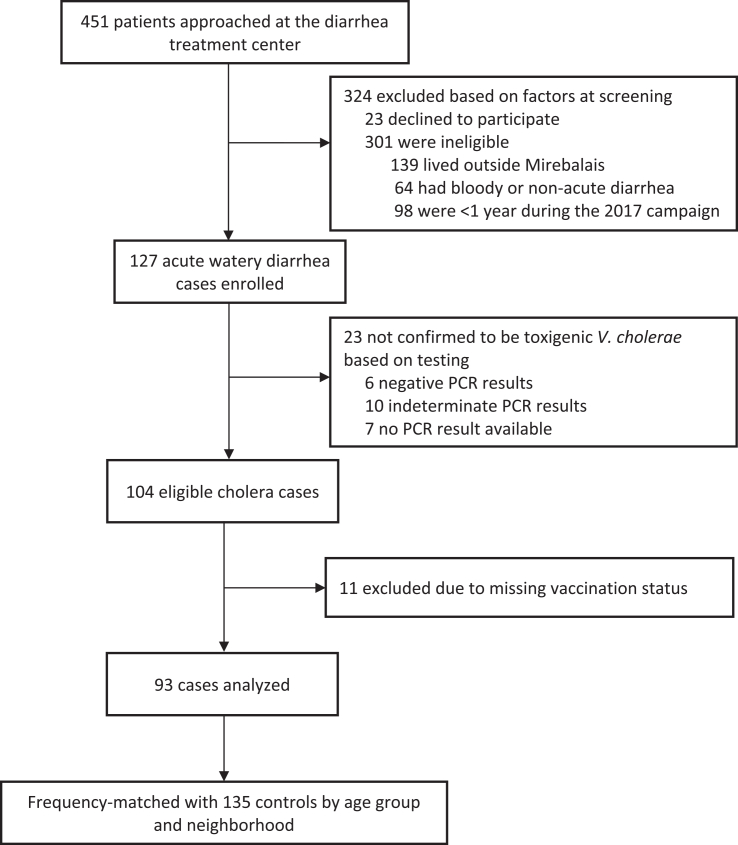
Enrollment of cases and controls in a 5-year study of oral cholera vaccine effectiveness (Euvichol®) in rural Haiti, Oct–Dec 2022.

**Fig. 2:**
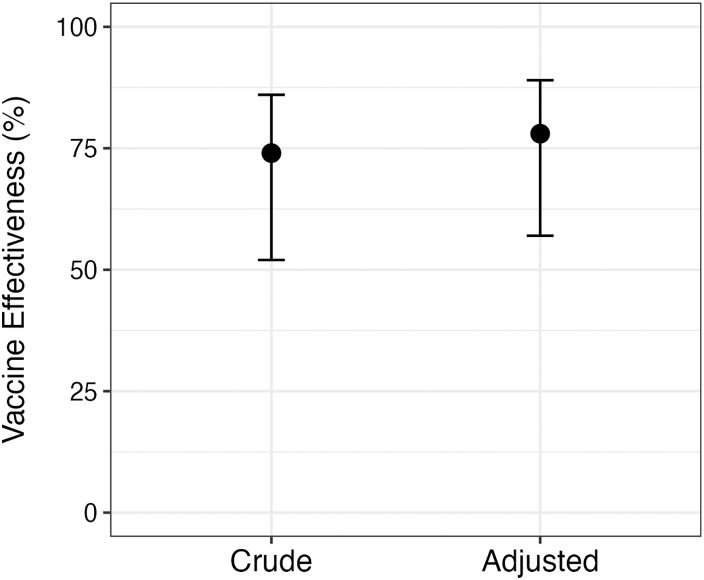
Crude and adjusted estimates of vaccine effectiveness in rural Haiti, 5 years after vaccination with a killed oral cholera vaccine (Euvichol®).

Among 451 patients approached at a diarrhea treatment for screening and inclusion as cases, we excluded 324 who did not meet our inclusion criteria, including 23 who declined participation and 301 who either lived outside of Mirebalais, had bloody or non-acute diarrhea, or were ineligible for OCV in 2017 (Fig. 1). We enrolled and tested 127 individuals by qPCR and excluded 23 who were not confirmed to have toxigenic *V. cholerae* (i.e., 6 PCR-negative, 10 PCR-indeterminate, and 7 with no result available). Of 104 PCR-confirmed cholera cases, we excluded 11 due to missing information on vaccination status. We analyzed data for 93 cases and 135 controls (Table 1). Most cases and controls were from the Grand Boucan communal section (65% for cases and controls). The median age among cases was 23 years old (interquartile range [IQR], 9–38) and among controls 27 years (IQR, 12–43). 20 of 93 cases (22%) and 69 of 135 controls (51%) reported vaccination. We were able to confirm vaccination status with a vaccine card for very few participants (seven total; one case and six controls). Within this group, there was only one instance of discordance in the number of doses received: the self-reported dose was “unknown”, but the documented dose was “one”. The odds ratio for any vaccination in 2017, adjusting for matching factors (age and neighborhood), was 0.24 (95% confidence interval [CI] 0.13–0.45) and adjusting for matching factors and confounders, was 0.22 (95% CI 0.11–0.43) (Supplementary Tables S2 and S3). Any kOCV dose received in 2017 was associated with a 5-year adjusted VE of 78% (95% CI 57%–89%, p < 0.0001) against medically-attended cholera in 2022 (Fig. 2). In 24 cases and 24 controls aged <5 years during the 2017 OCV campaign, 5 (21%) and 16 (67%) were vaccinated, respectively, and the 5-year VE, adjusting for neighborhood, was 88% (95% CI 50%–97%, p = 0.003) (Supplementary Tables S1 and S4). Sensitivity analyses in which we included only the 53 controls enrolled within ten days of the last case yielded similar vaccine effectiveness estimates to those derived in the primary analyses (81%; 95% CI: 56%–92%, p = 0.0001; Supplementary Table S5).

## Discussion

Euvichol® effectively protected against cholera during an outbreak of cholera in central Haiti, 5 years after a mass vaccination campaign. Our findings are consistent with the 5-year efficacy reported for those over 1 year old in a clinical trial of two doses of Shanchol® in an urban slum in Kolkata, India (VE 65%, 95% CI 52–74%; p < 0.0001), as well as with our group's 4-year effectiveness study of Shanchol® in Haiti (VE 76%, 95% CI 59–86; p < 0.0001).^3^^,^^4^ For Euvichol, we have previously shown a two-dose VE of 69% in Haiti at two years. Our study adds to the previously available data because it is the first long-term effectiveness data for Euvichol®, a major component of the global stockpile of kOCV.^15^ Our findings further add to the evidence because the epidemiologic context of Haiti is different than India, where the previous clinical trial took place. In Haiti, in 2017 at the time of the kOCV campaign, a major epidemic of cholera was still underway, however in the subsequent years, cholera cases waned such that between 2019 and 2022 no cases of cholera were detected in the country at all.^16^ As a result, risk of natural exposure to *V. cholerae* in the community in the years before the 2022 outbreak is unlikely to have contributed to shaping the immune response (‘immune boosting’) or the effectiveness we observed in our study. This is in contrast to VE studies in highly endemic regions. In India, cholera is more widespread and efficacy estimates in the aforementioned study thereby include protection from both vaccine-induced immunity and immunity induced by natural cholera infections occurring after the participants received vaccine or placebo. For example, a large outbreak of cholera in the 4th year of that study may have been a factor in the higher VE in year 5 than in year 4. However, natural immunity may also have been increasing over time among the unvaccinated in the study, which would bias VE toward the null.^3^

Our finding that Euvichol® offered protective effectiveness against cholera 5 years after a campaign in Haiti supports the use of kOCV as part of comprehensive cholera control and prevention, and contributes to considerations regarding booster regimens in epidemic-prone areas.^17^ Cholera vaccines are currently supported to countries through two supply mechanisms, both of which are funded at least in part by Gavi, The Vaccine Alliance. Although there are no formal recommendations on boosting regimens, Gavi's 2018 investment case and 2023 Market Shaping Roadmap consider kOCV campaigns as having protection of “approximately 3 years”.^17^^,^^18^ In 2017, using then-available evidence, WHO's Strategic Advisory Group of Experts (SAGE) recommended that follow-up kOCV campaigns “may be considered” after 3 years.^19^ While studies investigating the immunogenicity of vaccination schedules that either delay the second dose of kOCV by weeks or months, administer a booster dose three years after single-dose vaccination, or provide a booster dose three years after initial series for children under five years old,^20^^,^^21^ offer immune insights for primary recommendations, our study suggests that clinical protection from medically-attended diarrhea is afforded for as long as 5 years after a primary kOCV series in an epidemic-prone setting.

Our findings should be interpreted in the context of several limitations. First, people with and without diarrhea may differ in ways other than vaccination, including exposure to cholera, practices, and access to safe drinking water and sanitation. We sought information on these risk factors and while we cannot rule out unmeasured confounding, cases and controls were generally similar across most measured characteristics and adjustment for potential confounders did not notably change the vaccine effectiveness estimate. Second, most controls were recruited after case enrollment was complete, coinciding with the start of a new single dose kOCV campaign. We took great care to ensure that 2017 was the temporal reference point for self-reported vaccination. We undertook the aforementioned sensitivity analysis restricted to controls enrolled within ten days of the last enrolled case and this demonstrated findings that were consistent with primary analyses. Third, the vaccination status of adults and children is likely positively correlated within a household; thus, the apparent protective association in children, which is higher than reported direct protective efficacy in clinical trials, may reflect decreased risk of exposure to cholera within vaccinated households, rather than direct vaccine protection. For example, one study found that maternal receipt of OCV was associated with a 47% reduction in cholera among their non-vaccinated children under 36 months of age. However, we did not have measures of household vaccination coverage to test this hypothesis.^22^ Finally, given the limitations of self-reported vaccination and the extended vaccination recall period of five years, we did not attempt to distinguish between the effectiveness of one and two doses in 2017 on medically-attended cholera in 2022.

Whereas access to safe water, food, as well as sanitation and hygiene are critical components of controlling and eliminating cholera, kOCV is an important tool and our study suggests that effectiveness in an epidemic-prone context extends to at least 5 years. This study adds to the discussion of the role of kOCV in long-term control of cholera in certain settings and allows comparative analysis of different long-term strategies. Additional research on the long-term immunogenicity and clinical effectiveness of kOCVs is needed across dosing strategies, age groups, and epidemiologic settings to ensure their deployment with maximal impact.^15^

## Contributors

LCI conceived the study, co-designed the protocol, contributed to analysis and interpretation of data, and wrote the first draft of the manuscript. GCA, AC, YG led data collection, data management, and participated in data interpretation. RT contributed to the study design and data interpretation. JBH and DMS contributed to data analysis and lead specimen analysis and interpretation. MFF co-designed the protocol, analyzed the data, and contributed to data interpretation. AL contributed to data analysis. All authors contributed to manuscript revisions and approved the final version for publication.

## Data sharing statement

De-identified participant data will be made available in Harvard Dataverse after publication of this article.

## Declaration of interests

The authors declare no conflict of interest. Euvichol ® was delivered as part of a public health activity managed by the Ministry of Health of Haiti in 2017. The non-profit organization Zanmi Lasante has been engaged in cholera control and prevention activities, including vaccination activities against cholera since 2010 in the region being studied. Some authors (LCI, YG, RT) have received funding for comprehensive cholera control programs and research activities in the past, but not from vaccine manufacturers. LCI was a member of the SAGE Working Group on Oral Cholera Vaccines (2015–2017) which reviewed research and evidence on oral cholera vaccines and proposed recommendations to SAGE.

## References

[1] Baik Y.O., Choi S.K., Olveda R.M. (2015). A randomized, non-inferiority trial comparing two bivalent killed, whole cell, oral cholera vaccines (Euvichol vs Shanchol) in the Philippines. Vaccine.

[2] Odevall L., Hong D., Digilio L. (2018). The Euvichol story – development and licensure of a safe, effective and affordable oral cholera vaccine through global public private partnerships. Vaccine.

[3] Bhattacharya S.K., Sur D., Ali M. (2013). 5 year efficacy of a bivalent killed whole-cell oral cholera vaccine in Kolkata, India: a cluster-randomised, double-blind, placebo-controlled trial. Lancet Infect Dis.

[4] Franke M.F., Ternier R., Jerome J.G., Matias W.R., Harris J.B., Ivers L.C. (2018). Long-term effectiveness of one and two doses of a killed, bivalent, whole-cell oral cholera vaccine in Haiti: an extended case-control study. Lancet Glob Health.

[5] Ali M., Qadri F., Kim D.R. (2021). Effectiveness of a killed whole-cell oral cholera vaccine in Bangladesh: further follow-up of a cluster-randomised trial. Lancet Infect Dis.

[6] Xu H., Tiffany A., Luquero F.J. (2025). Protection from killed whole-cell cholera vaccines: a systematic review and meta-analysis. Lancet Glob Health.

[7] Matias W.R., Guillaume Y., Cene Augustin G. (2024). Effectiveness of the Euvichol® oral cholera vaccine at 2 years: a case-control and bias-indicator study in Haiti. Int J Infect Dis.

[8] Pierre K. (2018). Ministère de la Santé Publique et de la Population (MSPP), Haiti. Use and perspectives of the oral cholera vaccine (ocv) as a component of the cholera response. https://www.gtfcc.org/wp-content/uploads/2020/08/5th-gtfcc-meeting-2018-katilla-pierre-haiti.pdf.

[9] World Health Organization (2005). https://iris.who.int/handle/10665/43209.

[10] Edmond Y.M. (2007). The lakou system : a cultural, ecological analysis and mothering in rural Haiti. https://media.mouka.ht/document/lakou-system-cultural-ecological-analysis-and-mothering-rural-haiti.

[11] Franke M.F., Jerome J.G., Matias W.R. (2017). Comparison of two control groups for estimation of oral cholera vaccine effectiveness using a case-control study design. Vaccine.

[12] Azman A.S., Parker L.A., Rumunu J. (2016). Effectiveness of one dose of oral cholera vaccine in response to an outbreak: a case-cohort study. Lancet Glob Health.

[13] Guillaume Y., Debela M., Slater D. (2023). Poor sensitivity of stool culture compared to polymerase chain reaction in surveillance for vibrio cholerae in Haiti, 2018-2019. Open Forum Infect Dis.

[14] Liu J., Gratz J., Amour C. (2016). Optimization of quantitative pcr methods for enteropathogen detection. PLoS One.

[15] Gavi, the Vaccine Alliance Meet the global oral cholera vaccine stockpile. https://www.gavi.org/vaccineswork/meet-global-oral-cholera-vaccine-stockpile.

[16] World Health Organization (2022). Cholera - Haiti. https://www.who.int/emergencies/disease-outbreak-news/item/2022-DON427.

[17] Gavi, the Vaccine Alliance (2018). Oral cholera investment case. Vaccine investment strategy - programme and policy committee meeting. https://www.gavi.org/sites/default/files/document/ppc-meeting-18-19-october-2018---vis-06a---annex-c--oral-cholera-investment-casepdf.pdf.

[18] Gavi, the Vaccine Alliance (2023 May). Gavi Alliance market shaping roadmap for oral cholera vaccines. https://www.gavi.org/sites/default/files/about/market-shaping/roadmaps/Cholera-RM_Public-Summary.pdf.

[19] World Health Organization (2017 Mar). https://terrance.who.int/mediacentre/data/sage/SAGE_Docs_Ppt_Apr2017/5_session_cholera/Apr2017_session5_oral_cholera_whole-cell-killed.pdf.

[20] Chowdhury F., Bhuiyan T.R., Akter A. (2020). Augmented immune responses to a booster dose of oral cholera vaccine in Bangladeshi children less than 5 years of age: revaccination after an interval of over three years of primary vaccination with a single dose of vaccine. Vaccine.

[21] Kanungo S., Desai S.N., Saha J. (2015). An open label non-inferiority trial assessing vibriocidal response of a killed bivalent oral cholera vaccine regimen following a five year interval in Kolkata, India. PLoS Negl Trop Dis.

[22] Clemens J.D., Sack D.A., Harris J.R. (1988). Impact of B subunit killed whole-cell and killed whole-cell-only oral vaccines against cholera upon treated diarrhoeal illness and mortality in an area endemic for cholera. Lancet.

