# Supplementary material for: 5-Year effectiveness of an oral cholera vaccine in a cholera outbreak in rural Haiti: a case–control study
Source: Lancet Reg Health Am. 2026 May 30;60:101500. doi: 10.1016/j.lana.2026.101500 (PMC13241657; doi:10.1016/j.lana.2026.101500)
Supplement: Supplementary Tables [file mmc1.pdf]

Supplementary Table 1. Estimates of effectiveness of kOCV in rural Haiti after 5 years, children less than 10 years of age at enrollment (<5 years of age during the 2017 campaign; N=48; 24 cases, 24 controls)

	**Cases**<br>**n (%)**<br>**N=24**	**Controls**<br>**n (%)**<br>**N=24**	**RR***<br>**[95% CI]**	**Vaccine**<br>**effectiveness***<br>**[95% CI]**	**p-value**
Vaccinated (any), self-report	5 (20.8)	16 (66.7)	0.12 [0.03, 0.50]	88% [50% – 97%]	0.003

*adjusted for neighborhood

Supplementary Table 2. Logistic regression model output (adjustment for matching factors only), primary analysis (N=228; 93 cases; 135 controls)

Variable	OR (95% CI)	p-value
Received cholera vaccine	0.24 (0.13, 0.45)	<.0001
Grand Boucan communal section*	0.56 (0.22, 1.47)	0.42
Sarazin communal section*	0.53 (0.18, 1.52)	0.36
Age (continuous)	0.99 (0.98, 1.00)	0.16old

* relative to Crête Brûlée and Gascogne;

Supplementary Table 3. Logistic regression model output (adjustment for matching factors and confounders), primary analysis (N=228; 93 cases; 135 controls)

Variable	OR (95% CI)	p-value
Received cholera vaccine	0.22 (0.11, 0.43)	<0.0001
Grand Boucan communal section*	0.82 (0.26, 2.61)	0.74
Sarazin communal section*	0.68 (0.19, 2.40)	0.55
Ever attended school	2.95 (1.23, 7.08)	0.016
Female sex	0.68 (0.36, 1.26)	0.22
Electricity in the home	0.77 (0.39, 1.49)	0.44
Household contact history of spending a night in a cholera treatment unit	2.47 (1.01, 6.04)	0.049
Frequency of handwashing (0 times / day)**	2.14 (0.38, 12.17)	0.39
Frequency of handwashing (1 times / day)**	0.64 (0.24, 1.68)	0.36
Frequency of handwashing (2 times / day)**	2.06 (0.90, 4.71)	0.09
Frequency of handwashing (3 times / day)**	1.01 (0.43, 2.35)	0.99
History of household diarrhea in the past two weeks	3.64 (1.26, 10.52)	0.017
Age (continuous)	1.00 (0.99, 1.02)	0.62
Household size (continuous)	0.99 (0.88, 1.11)	0.86
Agriculture as main income generating activity	1.04 (0.52, 2.09)	0.91

* relative to Crête Brûlée and Gascogne; **relative to 4 or more times per day

Supplementary Table 4. Logistic regression model output, (adjustment for neighborhood), subgroup analysis in children less than 10 years of age at enrollment (<5 years of age during the 2017 campaign; N=48; 24 cases, 24 controls)

**Variable**	**OR (95% CI)**	**p-value**
Received cholera vaccine	0.12 (0.03, 0.50)	0.003
Grand Boucan communal section*	0.87 (0.08, 9.11)	0.91
Sarazin communal section*	0.71(0.04,12.68)	0.81

* relative to Crête Brûlée and Gascogne

Supplementary Table 5. Logistic regression model output (adjustment for matching factors and confounders), sensitivity analysis in which we included only the controls enrolled within ten days of the last case (N=146; 93 cases, 53 controls)

**Variable**	**OR (95% CI)**	**p-value**
Received cholera vaccine	0.19 (0.08, 0.44)	0.0001
Grand Boucan communal section*	1.10 (0.43, 2.86)	0.84
Ever attended school	2.92 (0.92, 9.26)	0.07
Female sex	1.19 (0.52, 2.70)	0.68
Electricity in the home	1.24 (0.51, 3.03)	0.63
Household contact history of spending a night in a cholera treatment unit	3.46 (0.99, 12.11)	0.052
Frequency of handwashing (0 times / day)**	2.58 (0.29, 22.77)	0.39
Frequency of handwashing (1 times / day)**	0.54 (0.17, 1.67)	0.29
Frequency of handwashing (2 times / day)**	2.66 (0.86, 8.21)	0.09
Frequency of handwashing (3 times / day)**	2.02 (0.64, 6.35)	0.23
History of household diarrhea in the past two weeks	1.14 (0.33, 3.93)	0.83
Age (continuous)	1.01 (0.99, 1.04)	0.36
Household size (continuous)	1.00 (0.86, 1.17)	0.97
Agriculture as main income generating activity	0.80 (0.33, 1.96)	0.63

* relative to Crête Brûlée, Gascogne, and Sarazin; **relative to 4 or more times per day
